# Editorial: From randomized clinical trials to real-world data and big data sciences: Generating evidence-based medicine for value in western and herbal medicines

**DOI:** 10.3389/fpubh.2023.1129399

**Published:** 2023-02-13

**Authors:** Xiaomo Xiong, Gang Lv, Bin Jiang, Kevin Lu

**Affiliations:** ^1^Department of Clinical Pharmacy and Outcomes Sciences, University of South Carolina, Columbia, SC, United States; ^2^Department of General Surgery, The First Medical Center of Chinese PLA General Hospital, Beijing, China; ^3^Department of Administrative and Clinical Pharmacy, School of Pharmaceutical Sciences, Health Science Center, Peking University, Beijing, China

**Keywords:** real-world data, randomized controlled trial (RCT), evidence based medicine, value in health, herbal medicine, Traditional Chinese Medicine

Evidence-based medicine (EBM) differs from traditional medical practices in that it places emphasis on the use of published evidence and rigorous evaluation (1). The main idea of EMB is that medical decisions should be based on objective research findings as much as possible (1, 2), and EBM is increasingly accepted in clinical practice and has a wide range of implementation (3). The goal of this Research Topic is to provide evidence-based medicine by assessing the effectiveness, safety, and economics of medicines that matter to patients, where we have collected a series of studies related to the topic. The collected studies under this topic cover a wide range of evidence-based studies related to clinical effectiveness, including comparative effectiveness research using real-world data, research related to the coronavirus disease of 2019 (COVID-19) pandemic, and systematic reviews and meta-analysis to summarize the best evidence-based medicine available in the literature. We expect that the research on this topic or the evidence they provide will act an informative role in clinical practice, to guide clinical treatment or formulate intervention measures.

Most clinical interventions that are marketed or approved have undergone a rigorous process of the gold standard of EBM, i.e., the randomized controlled trial (RCT), to measure clinical efficacy (4–7). However, in RCTs, the setting is rigorous, and the strict inclusion and exclusion criteria for patients may affect the external study of their findings (6, 8). Therefore, evidence derived from real-world studies or clinical effectiveness is warranted to guide day-to-day clinical practice (9). Meanwhile, many alternative treatments, such as herbal medicine and traditional medicine, are used in clinical practices, however, with a lack of evidence on efficacy from RCTs (10). As such, evidence of their clinical effectiveness is crucial. Therefore, being able to obtain more evidence about these studies is of great significance for informing practice.

Although EBM informing clinical practice and public health policy decision-making is well-documented in the literature, the role of EBM in some aspects of clinical effectiveness, such as clinical trial designs, medication use, quality of life, and the economic burden has received less attention. Several articles under this Research Topic may complement this point. First, for clinical trial designs, Iglesias-Lopez et al. conducted a systematic review, which found that most authorized advanced therapy medicinal products (ATMPs) are based on small, open-label, uncontrolled, and single-arm trials. The findings of this research suggest that in future studies, more methodologically sound randomized-controlled trials for ATMPs are needed. Meanwhile, a case study by Rui et al. found a relationship between short-term surrogate endpoint indicators and median progression-free survival (mPFS) and median overall survival (mOS) in clinical trials of malignant tumors. Second, for medication use, based on the hospital database with 3,422,710 outpatient and emergency visits and 26, 118, 436 inpatient hospitalizations from 2016 to 2019, Zhu et al. studied the trends and patterns of use of antibiotics in China, which might help inform clinical guidance in the future. Finally, in terms of quality of life and economic burden, Zou H. et al. systematically reviewed the economic burden and quality of life of patients with hepatocellular carcinoma (HCC) in Greater China, and found that HCC had a negative impact on the quality of life among the patients, mainly related to the aspects of physical, cognitive, and social functions. At the same time, this study revealed the impact of HCC on the economic burden of patients, which showed that HCC significantly increased patients' health-related costs (Zou H. et al.).

As the highest-level evidence in EBM that is able to integrate the results of multiple studies, meta-analysis can implement the generalization of RCTs and enhance the guidance of medical practice (11, 12). This Research Topic includes several systematic reviews and studies of meta-analysis that can help inform clinical practice. Specifically, these studies provide clinical evidence for several chronic conditions. Chen X. et al. systematically reviewed the clinical efficacy of conservative treatments on lumbar spinal stenosis, which found that limaprost might have better efficacy. In another review about the safety of fostamatinib among patients with rheumatoid arthritis, Chen Y. et al. found that compared to the placebo, fostamatinib was associated with a higher risk of malignant neoplasms at 52 weeks, with an odds ratio of 4.49 (95% CI 1.03–19.60). In a study with more than 1,000 patients with Peyronie's disease by Cao et al., the authors found that collagenase clostridium histolyticum has a significant effect on treating Peyronie's disease. In addition to chronic diseases, this topic has collected several studies on evidence generated in systematic reviews for patients with cancer. Based on five trials with more than 3,000 patients, Chen J. et al. found that cabazitaxel is effective in post-docetaxel settings but associated with a high risk of serious adverse events (SAEs). Using meta-analysis with 13 studies, Zou X. et al. found that a poorer socioeconomic status was associated with an increased risk of lung cancer in adulthood, with an OR of 1.25 (95% CI 1.10–1.43).

In the context of the pandemic, this Research Topic also collects a study about the evidence on the effectiveness of using oral anticoagulation for coronavirus disease 2019 (COVID-19). Specifically, Dai et al. conducted a systematic review and meta-analysis and found that chronic oral anticoagulation could not reduce mortality or intensive care unit (ICU) admission rate for patients with COVID-19. This Research Topic also contains a research protocol for a further clinical trial to assess the efficacy of integrative acupuncture and moxibustion treatment in patients with major depressive disorder (MDD; Zhang et al.).

We believe that the studies included in this Research Topic can serve as a clinical guide to a certain extent, especially as a supplement to randomized clinical trials. At the same time, we also believe that these studies can serve as a methodological inspiration for future research. Ultimately, we hope this Research Topic can support research on the value of health in evidence-based medicine.

## Author contributions

KL, XX, GL, and BJ conceived the idea for the editorial and wrote the initial draft. All authors approved the final version of the editorial.
